# Supplementary material for: Assessing Patient Understanding and Adherence to Preoperative Medication Advice Provided in Pre-Admission Clinic
Source: Healthcare (Basel). 2025 Sep 25;13(19):2429. doi: 10.3390/healthcare13192429 (PMC12524066; doi:10.3390/healthcare13192429)
Supplement: Supplementary file 1 [file healthcare-13-02429-s001.zip › healthcare-3841390-supplementary.pdf]

Table S1: Participant Survey

Prior to obtaining consent and conducting the patient survey, investigator to review the electronic medical record and determine how medication advice was provided, which medicines required perioperative management, and what instructions were given to the patient.		
**Q1**	**You were given some medication instructions *[in person/by phone/by SMS/by email]* before the surgery. Can you tell me what you were told to do about your *[medication requiring perioperative management]*?**	
	Investigator - Did the patient correctly recall all medication advice?<br>[ ] Yes [ ] No	
**Q2**	**How did you take your *[medication requiring perioperative management]*?**	
	Investigator - Record how each medication was taken and the reason(s) for non-adherence to medication advice. Patient is adherent if all medication advice was correctly followed.<br>[ ] Adherent [ ] Non-adherent	
	**Aspirin**<br>[ ] Stopped on DOS<br>[ ] Stopped too early (before DOS)<br>[ ] Stopped too late	**NSAID**<br>[ ] Stopped on DOS<br>[ ] Stopped too early (before DOS)<br>[ ] Stopped too late<br>(if used PRN, stopped too early or stopped too late is not applicable)
	**Clopidogrel/ticagrelor**<br>[ ] Stopped on DOS<br>[ ] Stopped too early (before DOS)<br>[ ] Stopped too late<br>[ ] Did not stop and bridge as advised	**DOAC/warfarin**<br>[ ] Stopped on DOS<br>[ ] Stopped too early (before DOS)<br>[ ] Stopped too late<br>[ ] Did not stop and bridge as advised
	**DMARD/biologic**<br>[ ] Stopped too early<br>[ ] Stopped too late	**Insulin**<br>[ ] Stopped too early<br>[ ] Stopped too late<br>[ ] Different dose administered
	**Oral hypoglycaemics (excluding SGLT2i)**<br>[ ] Stopped too early<br>[ ] Stopped too late	**SGLT2i**<br>[ ] Stopped too early<br>[ ] Stopped too late
	**GLP-1/GIP receptor agonist**<br>[ ] Stopped too early<br>[ ] Stopped too late	
**Q3**	**Do you think the instructions provided were clear and easy to understand?**	
	[ ] Yes [ ] No [ ] Do not remember	
**Q4**	***(If non-adherent)* The way your medications were managed is slightly different to the instructions you were given. Can you tell me the reason for this?**	
	[ ] Could not remember the instructions given<br>[ ] Did not understand, or misunderstood, the instructions<br>[ ] Medication changes in the dose administration aid were not made by the local pharmacy or by the nursing home<br>[ ] Conflicting instructions were received from multiple providers<br>[ ] Self stopped medications intentionally due to surgery<br>[ ] Other	
**Q5**	**What is your preference for the way medication instructions are communicated - verbal only, written only, or a combination of both verbal and written instructions?**	
	[ ] Verbal only [ ] Written only [ ] Combination of both verbal and written	
**Q6**	**Do you have any other comments about the instructions you were provided, or about your medication management before surgery?**	

DMARD = disease-modifying anti-rheumatic drug, DOAC = direct oral anticoagulant, DOS = day of surgery, GIP = gastric inhibitory polypeptide, GLP-1 = glucagon-like peptide-1, NSAID = non-steroidal anti-inflammatory drug, SLGT2i = sodium-glucose co-transporter-2 inhibitor
